# Performance of a Low Energy Ion Source with Carbon Nanotube Electron Emitters under the Influence of Various Operating Gases

**DOI:** 10.3390/nano10020354

**Published:** 2020-02-18

**Authors:** Huzhong Zhang, Detian Li, Peter Wurz, Adrian Etter, Yongjun Cheng, Changkun Dong, Weijun Huang

**Affiliations:** 1 Science and Technology on Vacuum Technology and Physics Laboratory, Lanzhou Institute of Physics, Lanzhou 73000, China; janehuge@126.com (H.Z.); chyj750418@163.com (Y.C.); 2 Physics Institute, University of Bern, 3012 Bern, Switzerland; adrian.etter@space.unibe.ch; 3 Institute of Micro-Nano Structures & Optoelectronics, Wenzhou University, Wenzhou 325035, China; dck@wzu.edu.cn (C.D.); hwj@wzu.edu.cn (W.H.)

**Keywords:** ion source, electron field emission, gas adsorption, carbon nanotube

## Abstract

Low energy ion measurements in the vicinity of a comet have provided us with important information about the planet’s evolution. The calibration of instruments for thermal ions in the laboratory plays a crucial role when analysing data from in-situ measurements in space. A new low energy ion source based on carbon nanotube electron emitters was developed for calibrating the ion-mode of mass spectrometers or other ion detectors. The electron field emission (FE) properties of carbon nanotubes (CNTs) for H_2_, He, Ar, O_2_, and CO_2_ gases were tested in the experiments. H_2_, He, Ar, and CO_2_ adsorbates could change the FE temporarily at pressures from10^−6^ Pa to10^−4^ Pa. The FE of CNT remains stable in Ar and increases in H_2_, but degrades in He, O_2_, and CO_2_. All gas adsorbates lead to temporary degradation after working for prolonged periods. The ion current of the ion source is measured by using a Faraday cup and the sensitivity is derived from this measurement. The ion currents for the different gases were around 10 pA (corresponding to 200 ions/cm^3^ s) and an energy of ~28 eV could be observed.

## 1. Introduction

Detection of ions in-situ in the environment of a planetary body plays a crucial role in the investigation of a planet. The solar system’s evolution can be tracked by studying ions in space. For instance, “cold” ions in the vicinity of comet 1P/Halley has shown us a lot of information about cometary plasma and the interaction between comets and solar winds [1,2]. The Rosetta Orbiter Spectrometer for Ion and Neutral Analysis (ROSINA) experiment measured the volatile components of the cometary coma of comet 67P/Churyumov-Gerasimenko (C-G) [3]. Ion measurements from ROSINA have significantly improved our knowledge about the interaction of the comet with solar wind plasma. For the accurate measurement of ions or plasma in space, the precise calibration of instruments is required in the laboratory. A calibration facility for solar wind plasma instruments was built using an electron-cyclotron-resonance ion source operating at 2.45 GHz, which could test ions produced from elements ranging from gaseous to solid [4]. A calibration apparatus called SATANS (supersonic cation and anion source) was developed for calibrating a NIM (neutral gas and ion mass spectrometer) in ion modes ranging from 0.01 eV–30 eV [5,6,7], with respect to all JUICE (JUpiter ICy moons Explorer) mission requirements. A compact ion source was also constructed, based on microtips as electron field emitters for calibrating the ion measurement mode of the ROSINA instruments flying in the Rosetta mission [8]. For calibration of low energy ions in space [9], the challenge was mainly the production of a homogeneous ion beam at very low energies. Therefore, the potential differences in the ion source resulting from the electron emitter, materials, potential differences, etc., should not be neglected in ion calibration. The electron field emitter showed several advantages, i.e., narrower additional energy distribution for molecular gases due to the low temperature of the emitter, lower space charge of electrons due to the uniform spatial distribution from the surface of the emitter [10], and a higher power efficiency than a hot filament as well.

As an efficient electron field emitter, the carbon nanotube (CNT) is a promising candidate to be applied as a compact ion source because of its reliability and environmental compatibility. Even though a wide range of gases, including H_2_, CO_2_, CH_4_, NH_3_, CO_2_, and others, have been successfully detected by CNT-based sensors through the mechanisms of electron field emission (FE) [11], heat resistance [12], electrical resistance, etc. [13,14], carbon nanotube devices were thought to be immature for applications as an ion source due to the lack of understanding about the performance of a CNT emitter for different operating gases while it was applied as a standard ion source. In this paper, a low-energy ion source that originally used microtip electron field emitters [15] was re-designed to use CNT-based electron emitters to have a CNT-based low energy ion source (CNT-LEIS). The reaction of the CNT with different calibration gases in the ion source was studied. Simultaneously, the ion production and sensitivity of the ion source with different operating gases were investigated. Particularly, the FE current, electron beam stability, and FE reversibility of the CNT emitter were evaluated with different operating gases.

## 2. Layout of CNT-LEIS

**Figure 1:**
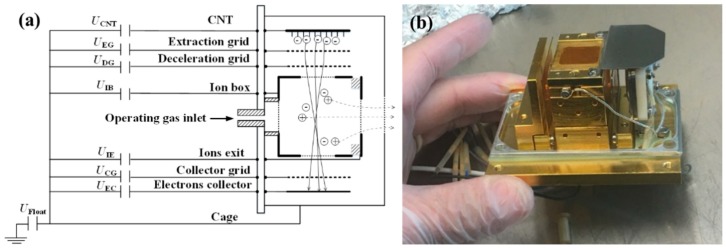
Schematic diagram (**a**) and photograph (**b**) of the low energy ion source with a carbon nanotube (CNT) electron emitter. The symbols ⊕ and ⊖ represent ions and electrons, respectively.

The CNT-LEIS is basically a classic electron ionization ion source. However, the ionization volume is maximized to avoid the space charge effecting the ion population, in order to reach lowest ion energies. A schematic diagram of the CNT-LEIS is shown in Figure 1a. The electrons are emitted from the CNT electron emitter via field emission induced by the extraction voltage applied to the extraction grid. The ions’ and electrons’ trajectories in the ion box are also shown schematically in Figure 1a. The electrons released from the CNTs pass the extraction grid, deceleration grid and the entrance of ion box to collide with gas molecules to produce ions in the ionization volume. The electrons continue their path, leave the ionization box, pass through the collector grid and are collected by the electron collector. The ions produced inside the ionization volume are extracted from the ion box orthogonal to the direction of the electron flow, because of a small voltage difference between the ion box, on potential *U*_IB_, and the ion exit grid, on potential *U*_IE_. The small potential difference between *U*_IB_ and *U*_IE_ of 0.3 V only weakly affects the trajectories of electrons with energies of around 70 eV, to keep the whole flight path of electrons in the ionization volume nearly field-free, but guides the ions to the exit aperture. Space charge by the electrons is negligible, and collisions among particles can be ignored. The final energy of ions leaving the ion source is given by the potential difference between the ion exit and the cage. The individual voltages of the ion source are set to *U*_IB_ = 10.2 V, *U*_IE_ = 9.9 V, *U*_float_ = 0 V, *U*_CG_ = 10 V, and *U*_EC_ = 50.5 V during these measurements. The CNT bias voltage *U*_CNT_ is fixed to -65 V to ensure a constant electron energy of around 75 eV, with the final electron energy being the potential difference between the CNT surface, *U*_CNT_, and the ion box *U*_IB_. The electron energy determines the fragmentation of the sample molecules in the process of electron impact ionization [16]. A photograph of the CNT-LEIS with the cage removed is illustrated in Figure 1b. The CNT emitter with a ceramic bracket is placed on the right side of picture. The exit of ions is on the top of the device, as seen in Figure 1b.

The CNT-LEIS can work in two modes, depending on different gas inlet systems [15]. In dynamic mode, the gas could be introduced through a glass capillary plate at the bottom of the picture shown in Figure 1b, in order to form a parallel gas flow of the working gas along the primary axis of the ion source, which is in the direction of ion extraction (vertical direction in Figure 1b). This configuration minimizes the initial energy spread of the gas in the ionization box perpendicular to the extraction direction. In static mode, the working gas is introduced into the vacuum chamber where the experiment was carried out, and this pressure is kept constant. The dynamic mode has been previously cross-calibrated with the use of the measured signal in static mode [15].

The CNT-LEIS, as electron impact ion source, produces ions at a ratio between the ion current *I*_i_ and the electron current *I*_e_, which was illustrated in Equation (1) [17].

(1) $$\frac{I_{i}}{I_{e}}=\frac{p_{i}}{k_{b}\cdot T}\cdot L\cdot \sigma_{i}(E)$$

In Equation (1), *E* is the kinetic energy of the electrons, *p*_i_ is the neutral gas pressure, *k*_b_ is the Boltzmann’s constant, *T* is the gas temperature, *L* is the ionization collision path length (depending on the electric filed distribution and the size of ionization chamber), and *σ*_i_(*E*) is the ionization cross-section for specific gas along the ionization collision path length. The sensitivity *S* is defined as

(2) $$S=\frac{L\cdot \sigma_{i}(E)}{K_{b}\cdot T}=\frac{I_{i}}{I_{e}\cdot p_{i}}$$

**Figure 2:**
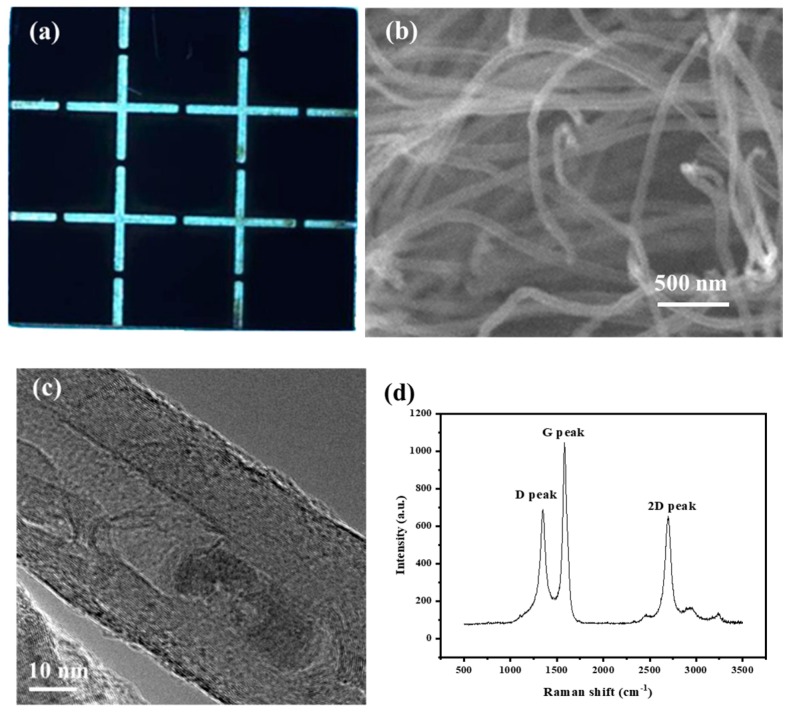
Characterization results of CNTs by optical microscope (**a**), SEM (**b**), TEM (**c**) and Raman spectroscopy (**d**).

In our experiment, as shown in Figure 2a, the total area of the electron emission of the CNTs was 11 × 11 mm^2^. The emission area of CNTs was grown as a 3 × 3 patterned structure (see Figure 2a) at Wenzhou University, China. CNTs with an average diameter around 40 nm–60 nm showed hollow and tubular structures (see Figure 2b). The surfaces of the CNTs were clean and smooth, without obvious amorphous carbon. The catalyst particles were embedded inside the CNT body, as shown in the TEM characterization in Figure 2c. The good crystallinity of CNTs can be inferred from the Raman spectrum (see Figure 2d). The intensity ratio of the G peak at 1582.2 cm^−1^ and D peak at 1351.7 cm^−1^, *I*_G_/*I*_D_, for the CNT, was about 1.5. The patterned emitter was supposed to enhance the field emission current from each single area of the CNT emitter, owing to the edge effect that the electric field was significantly higher at the edge than the center of the single area [18] electric field was significantly higher at the edge of the bundle than at the center. The CNTs were fixed in a ceramic bracket that kept the distance between the extraction mesh and CNTs at 0.4 mm. The extraction grid, made of molybdenum mesh with a transparency of 80%, was set to positive potential to extract electrons from the surface of the CNTs by electron field emission, according to the law of Fowler-Nordheim [19].

## 3. Properties of Electron Emitters in CNT-LEIS

### 3.1. Emission Properties of CNTs in Base Pressure

**Figure 3:**
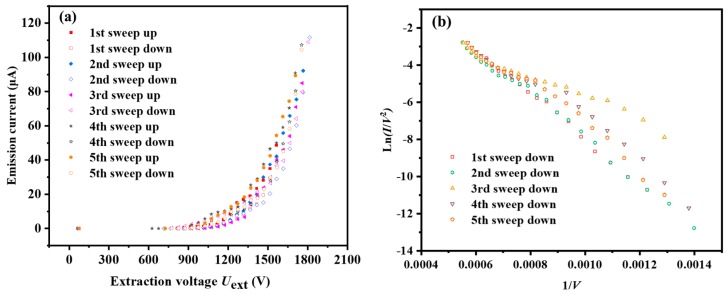
*I*-*V* property tests of multiple sweeps after 3 h of the conditioning process at a pressure of 1.2 × 10^−6^ Pa. (**a**) 5 times FE tests of up and down sweeps. (**b**) Ln(*I*/*V*^2^)-1/*V* curve plotted from *I*-*V* data of down sweeping.

The vacuum system for testing CNT-LEIS was baked at 80 °C for 96 h. The remaining residual gas (i.e., H_2_O, H_2_, CO, etc.) dominated the FE [20]. In Figure 3, the 1st and 2nd sweep (“up” and “down”) tests were conducted after 3 h conditioning without intervals. The current-voltage (*I*-*V*) tests from the 3rd to 5th sweeping up and down were carried out after an interval of 12 h when the residual gas, including H_2_O, H_2_ etc., was re-adsorbed on the CNTs again. An obvious hysteresis between “sweeping up” and “sweeping down” was observed, shown by the solid and empty symbols in Figure 3a. The FE of “down sweeps” corresponds to the relatively clean CNTs caused by joule heating during their operation. In addition, in Figure 3b, the Fowler-Nordheim (F-N) curve ln(*I*/*V*^2^) as function of (1/*V*) was plotted by “sweeping down” FE data to compare the linearity predicted for FE by the F-N theory. We find that 1st and 2nd sweeps are very close to the straight line representing typical FE emission from a quite clean surface of a CNT on account of conditioning. The other up and down sweeps deviate more from a straight line than the 1st and 2nd curves, due to re-adsorption of residual gas that changed the work function of CNT. From the 3rd to 5th sweeps, successive FE operations facilitate the desorption of residual gases and make the FE gradually approach the typical F-N curve.

### 3.2. Gas Adsorbates Influenced Field Emission

CNT-LEIS is applied to produce ions from the introduced atomic or molecular gas. Different working gases can be introduced into the ionization box through the glass capillary plate in dynamic mode. The effect on the electron beam by gas adsorbates on the CNTs has to be considered. That the adsorbates can influence FE has been illustrated before [21,22]. However, the effect of adsorbates varies significantly for CNTs in different working conditions. In the ion source, after been accelerated by the extraction grid and decelerated by the deceleration mesh as well as the ion box, etc., a fraction of the emitted electrons was collected by the electron collector of the ion source (see Figure 1). The collected electron current changed proportionally with the emission current. The variation of collected electrons, defined as the ratio of collected electron currents in corresponded partial pressure over that in base pressure, *r*, is used to evaluate the effect of different adsorbates.

**Figure 4:**
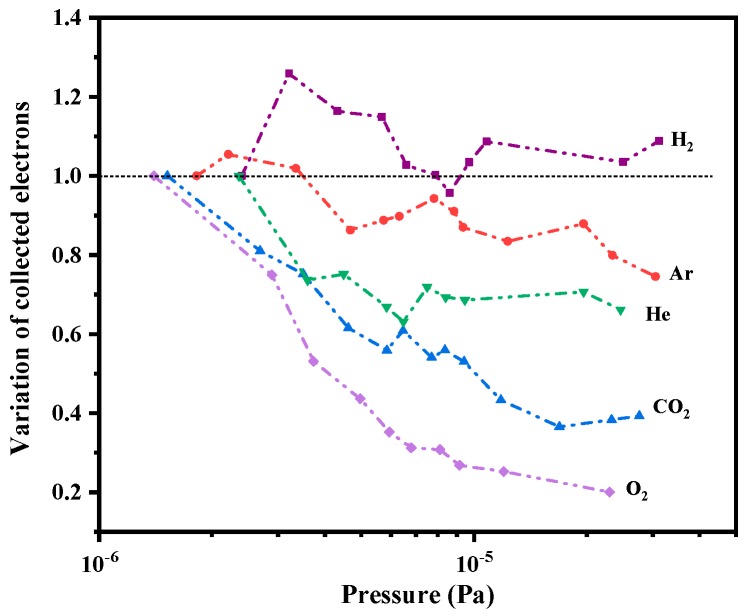
Emission variation caused by gas adsorbates.

Figure 4 shows the variation of collected current change with the pressure rise of chamber pressure by the operating gas. We found that the introduction of H_2_ caused a maximum collected current increase of 25%. This increase is attributed mainly to the C-H dipole-induced work function change of the CNTs. Such an influence has also been reported elsewhere [23,24]. The work function shift *ΔΦ* from the surface dipole layer can be evaluated from the relation [25]:

(3) $$\Delta \Phi \;=\;2\pi P_{i}N_{s}\theta$$

where *P_i_* is the dipole moment, *N_S_* is the maximum number of adsorption sites per unit area, and *θ* is the fraction of filled ones. The higher the gas partial pressure is, the larger the *θ*, leading to a bigger work function decrease.

In our experiment, we found that there is a small drop and then stability for H_2_ after a rapid increase in the FE current. In the ion source, the H_2_ adsorbates are removed by joule heating and achieve adsorption-desorption equilibrium along with the increase of H_2_ pressure, because H_2_ desorption could increase above temperatures of 600 K in high electric fields [26,27]. After removing H_2_, the inert gas Ar was introduced into the system. Since the residual H_2_ still occupies the adsorption sites at the beginning of introducing Ar as the operating gas, the slight decrease of FE ratio along Ar pressure is caused by the high FE current at an early stage of test. Comparatively, exposures with He, CO_2_, and O_2_ cause larger drops of the emission current than other gases. It was recognized that He, as an inert noble gas, should hardly influence the work function of CNTs [28]. In the experiment, the collected electron current for He as an operating gas at a pressure of ~10^−5^ Pa decreases about 35% and then stays stable. The bombardment of the CNTs by He^+^ at keV energy causes possible displacement of atoms in CNTs, leading to more desorption of H species because CNT samples irradiated with He^+^ indicated more damage compared to Ar^+^ irradiation, due to the much deeper penetration of He^+^ into the CNT [29,30]. The FE from the CNTs after the exposure of oxidized gases CO_2_ and O_2_, decreases 60% and 80%, respectively. Oxidative treatment is normally unfavorable to FE, with current degradation due to the work function shift or nanotube etching from chemical reactions [31]. The increased work function is the result of the combination of reduction of the p*π*-derived valence band DOS (density of states) and the presence of oxygen-induced surface dipole moments [20,32].

### 3.3. Reversibility of CNT Emissions in Different Gases

**Figure 5:**
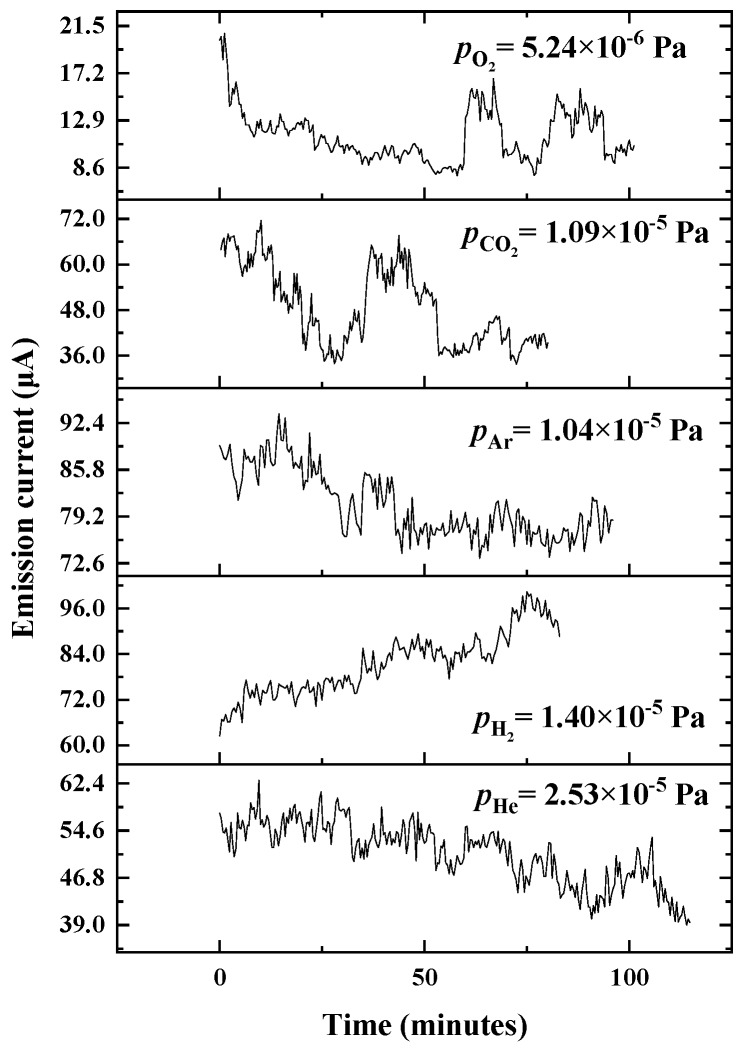
Emission current as function of operation time in He of 2.53 × 10^−5^ Pa, H_2_ of 1.4 × 10^−5^ Pa, Ar of 1.04 × 10^−5^ Pa, CO_2_ of 1.09 × 10^−5^ Pa, and O_2_ of 5.24 × 10^−6^ Pa.

**Figure 6:**
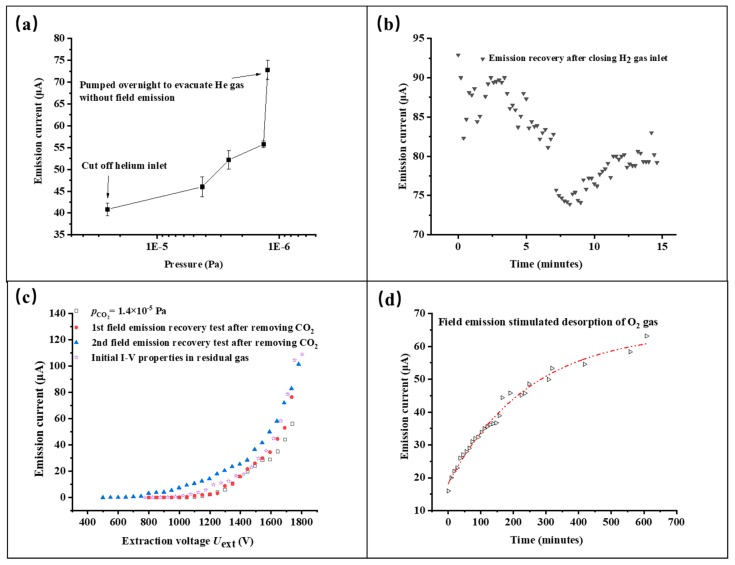
Reversibility of the electron field emission of CNT emitters after removing different operation gases, He (**a**), H_2_ (**b**), CO_2_ (**c**) and O_2_ (**d**), from the CNT-LEIS.

Carbon nanotubes, used as electron field emitters, show excellent reversibility from exposure to many sorts of gases. For CNT-LEIS, the typical continuous working period is always from several tens of minutes to a few hours [8]. We also evaluate the stability of the emitted electron current as the function of the working pressure, as well as reversibility after long-term tests in different ways, including degassing through pumping or FE stimulation. Before introducing the gases, the initial FE current is approximately 70 μA at an extraction voltage of 1750 V and a base pressure of 1 × 10^−6^ Pa. The FE properties for He, H_2_, Ar, CO_2_, and O_2_ were studied successively. Figure 5 shows the FE current variation after introducing He of 2.53 × 10^−5^ Pa. The initial current 70 μA decreased to 58 μA by sweeping up the extraction voltage from 0 V to 1750 V, and during the operation gradually reduces further to 40 μA in 110 min. By evacuating the remaining He gas overnight without FE operation, we find that the FE current is fully restored, which is shown in Figure 6a. Resulting from the low pumping speed of light He molecules for a turbomolecular pump in an ultrahigh vacuum [33], residual He gas in the chamber could decrease the FE slightly at the early stages of introducing H_2_. Figure 5 shows that FE is improved by 50% after operating the CNT emitter in an H_2_ gas of 1.4 × 10^−5^ Pa for 80 min, and then the current is restored in 10 min after closing the gas inlet, as shown in Figure 6b. A small increase of the FE current after 8 min indicates the re-adsorption of H_2_.

In Figure 5, due to the re-adsorption of H_2_, the initial FE current ~90 μA is far higher than original emission current when Ar is introduced in the CNT-LEIS following the H_2_ test. During operation in Ar, the FE decreases and stabilizes to about 77 μA after 50 min, approximately at the original electron emission. The result is different from well-known FE enhancement caused by Ar^+^ irradiation treatment at ~keV energy, which led to an increase of the field enhancement factor [34,35]. It indicates that Ar exposure has almost no influence on the FE current of CNTs, i.e., it does not change the work function. Similarly to the single-walled carbon nanotubes [20], Ar^+^ sputtering of the CNTs removed the re-adsorbed H_2_ molecules in 50 min, without any morphology damage.

As shown in Figure 5, gases with strong electronegativity, for example, CO_2_ and O_2_, weaken the FE current and lead to similar sudden changes in the period of the stability tests. Such kinds of decreases in FE current are mainly caused by surface chemical interactions, such as the formation of C-O dipoles [20]. Comparing two kinds of gases, the CNT emitter shows an extremely rapid drop with the increasing pressure of the O_2_ gas to 5.24 × 10^−6^ Pa after recovery from CO_2_-influenced FE. Some reports have explained it as a kind of ion bombardment-induced etching, especially at the surface, which preferentially removes the adsorbed H atoms from the CNT surfaces, and leads to a permanent decrease in the FE current [20,32]. However, we did not find a significant permanent degradation in 90–100 min of operation, showing the good FE recovery in Figure 6. Particularly, we employed different ways to restore the FE of CNT. For CO_2_, we conducted *I*-*V* tests in several sweeps to get rid of CO_2_ adsorbates by joule heating. As shown in Figure 6c, the 2nd restored curve after cutting off the CO_2_ gas is approximately coincident to the original *I*-*V* curve. Comparably, O_2_ was removed from the CNTs by long-term pumping and FE-stimulated degassing. We found that the emission current recovered almost to its nominal value from before, which is an increase from the emissions during O_2_ operation by 300%, as shown in Figure 6d. The C-O dipoles were possibly broken and the oxygen is desorbed during the FE operation in UHV (Ultrahigh vacuum), which was only found for single-walled carbon nanotubes and microtip array electron emitters [20,36].

## 4. Intensity and Sensitivity of CNT-LEIS

**Figure 7:**
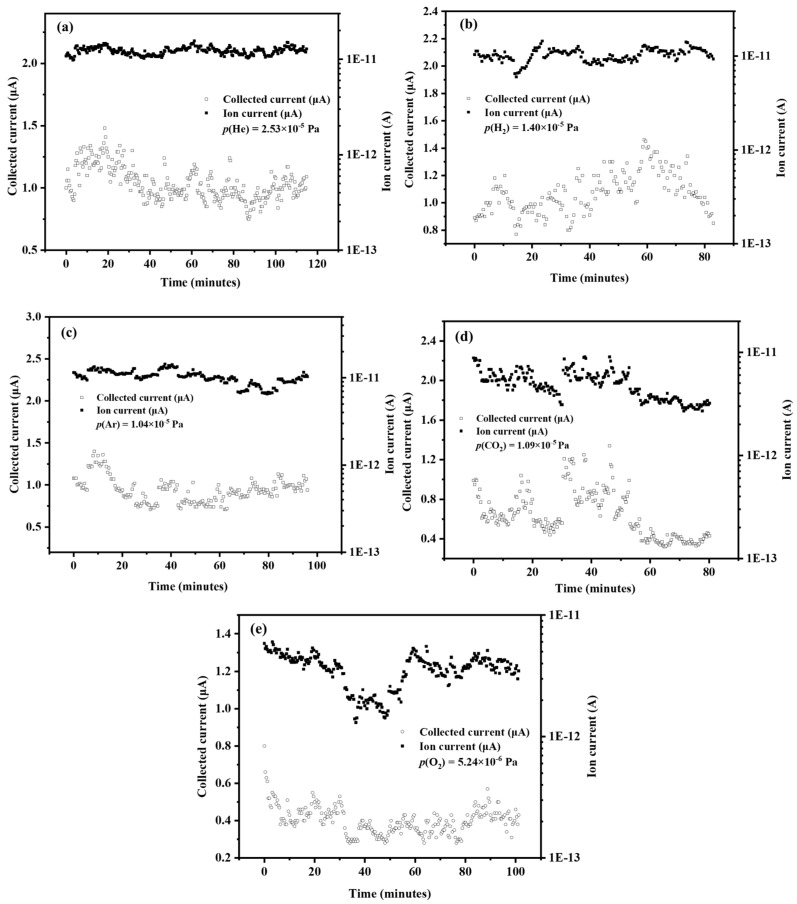
Ion current versus time for different gases, He (**a**), H_2_ (**b**), Ar (**c**), CO_2_ (**d**) and O_2_ (**e**), at the fixed extraction voltage.

The performance of the ion source was evaluated for different gases. The potential difference between the ion source and the external ion collector is −18 V. The ionization box is approximately 10 V. Although the electron emission current without regulation of the extraction voltage fluctuates a lot, as shown in Figure 5, the collected current stays relatively stable, which is the dominant factor for the reliability and stability of ion beams. The normalized ion current, defined as the ratio of ion current to the collected electron current, is an effective way to evaluate the ion beam stability versus time. Figure 7 shows the ion beam stability monitored by an external Faraday cup, located at about 2 cm from the exit of the ion source. A better ion current stability was obtained for He, H_2_, and Ar than that for CO_2_ and O_2_ as operating gases, while the electron extraction voltage is fixed at 1750 V. Oxidization of the CNTs (as shown Figure 4 and Figure 5) caused more fluctuation of the collected electron current, due to the formation and breaking of C-O dipoles. Moreover, the ion current for different gases was already around 10 pA, which corresponds to 200 ions/(cm^3^ s). In the application of the CNT-LEIS for calibration purposes, the collected electron current is stabilized, deploying regulating electronics of the extraction voltage. The ion current intensity and stability could be improved significantly.

**Figure 8:**
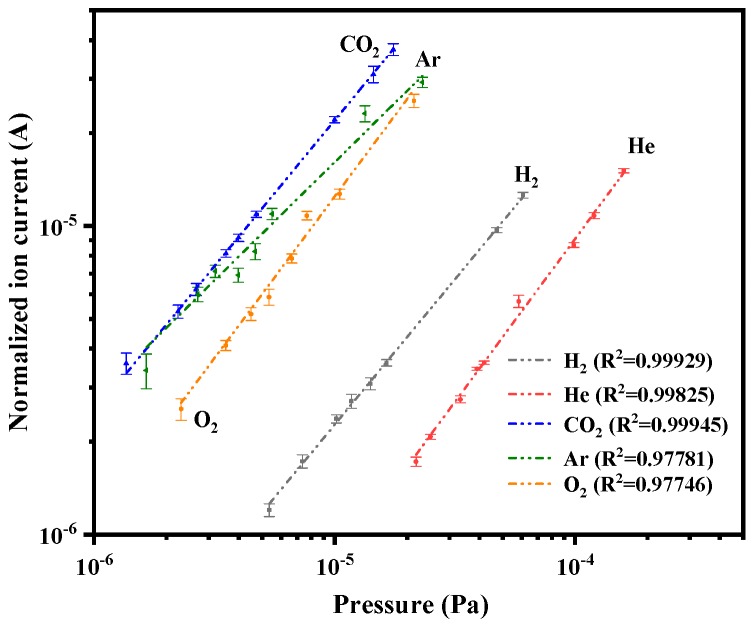
Linearity of the ion production of the CNT-LEIS for different operating gases.

To further evaluate the correlation between ion production rate and inlet gas pressure, the ion current was recorded along with the rise of inlet gas pressure. The inlet gas pressure, indicated by the ionization gauge at the wall of the vacuum chamber, was corrected using a scale factor for each gas of the gauge controller [37]. Thus, we use the corrected indicated pressures of H_2_, He, Ar, O_2_, and CO_2_ and the ion current normalized by collected current to calculate the sensitivity of CNT-LEIS by Equation (2). For all the gases, we could find that *I*_+_/*I*_e_ has a linear dependence on the pressure of the operating gas, as shown in Figure 8. The calculated sensitivity for different gases is recorded in Table 1. We found that the sensitivity of CO_2_ of 2.29 Pa^−1^, is the highest, while the sensitivity for He of 0.088 Pa^−1^ is the lowest. The relative value among all the calculated sensitivities, *S*(CO_2_) > *S*(Ar) > *S*(O_2_) > *S*(H_2_) > *S*(He), corresponds to the theoretical ionization cross-section for different gases, and the ion production rate fits the electron impact ionization model very well [16]. The relative standard deviation (RSD) of sensitivity for H_2_^+^, 5.1%, is the smallest. The sensitivity of Ar 1.93 Pa^−1^ with an RSD of 11.7% fluctuates more than other gases, which is probably caused by the changes of production and extraction efficiency for Ar^+^ from the exit of ion source. The production and extraction efficiency are always ionization cross-section and mass dependent. Considering the uncertainty of cross-sections of gases, the measurement RSD increases for larger ions (mass/charge).

## 5. Conclusions

We constructed CNT-based electron impact low-energy ion sources for the calibration of mass spectrometers for space applications. Custom-designed CNT electron emitters, as reliable electron field emitters, were applied to provide electrons to ionize a variety of gases for calibration purposes. The FE properties of CNT were evaluated comprehensively after a 3 h conditioning treatment. The CNT emitters showed excellent FE repeatability at a base pressure of the vacuum system of 1 × 10^−6^ Pa. The electron emission current varied with work function, morphology, etc., with different gases. Hydrogen enhanced the FE, but fell back to a stable value owing to adsorption-desorption equilibrium. However, for gases containing oxygen, like O_2_ and CO_2_, the CNT emitters showed significant degradation of the FE. For inert gases, we found that Ar did not change FE performance significantly but He led to a decrease of FE emissions, because of H desorption by deep penetration of He^+^ into CNTs. All the tested gases showed a temporary influence on the FE currents of the CNTs, increasing or decreasing the emitted electron current by rising the pressure of the inlet gas from 1 × 10^−6^ Pa to ~10^−4^ Pa. Moreover, the measured ionization data followed the electron impact ionization model very well. An ion current of 10 pA (corresponding to 200 ions/(cm^3^ s)), with an ion energy of ~28 eV, could be obtained in the current experiment. The stability of the ion beam of Ar^+^ (RSD of 11.7%) was worse than other that of gases, due to greater fluctuation of the extraction efficiency for larger ions (mass/charge). To make the ion source applicable for calibrating mass spectrometers, further tests of CNT-based ion sources in other gases will be conducted and evaluated. More suitable working pressures for calibration gases will also be investigated.

## Figures and Tables

**Table 1:** Calculated sensitivity of the ion current for different gases.

Gas	Sensitivity (Pa^−1^)	Relative Standard Deviation (RSD)
H_2_	0.221	5.1%
He	0.088	6.8%
CO_2_	2.30	6.5%
Ar	1.93	11.7%
O_2_	1.19	8.1%
